# Exploring the use of AI text-to-image generation to downregulate negative emotions in an expressive writing application

**DOI:** 10.1098/rsos.220238

**Published:** 2023-01-04

**Authors:** Gamar Azuaje, Kongmeng Liew, Rebecca Buening, Wan Jou She, Panote Siriaraya, Shoko Wakamiya, Eiji Aramaki

**Affiliations:** ^1^ Graduate School of Science and Technology, Nara Institute of Science and Technology, 8916-5 Takayama-cho, Ikoma, Japan; ^2^ Center for Research on End of Life Care, Weill Cornell Medicine, 1300 York Avenue, New York, NY 10065, USA; ^3^ Faculty of Information and Human Sciences, Kyoto Institute of Technology, Matsugasaki Hashikamicho, Sakyo Ward, Kyoto, Japan

**Keywords:** AI art, image generation, emotion regulation

## Abstract

Conventional writing therapies are versatile, accessible and easy to facilitate online, but often require participants to self-disclose traumatic experiences. To make expressive writing therapies safer for online, unsupervised environments, we explored the use of text-to-image generation as a means to downregulate negative emotions during a fictional writing exercise. We developed a writing tool, StoryWriter, that uses Generative Adversarial Network models to generate artwork from users’ narratives in real time. These images were intended to positively distract users from their negative emotions throughout the writing task. In this paper, we report the outcomes of two user studies: Study 1 (*N* = 388), which experimentally examined the efficacy of this application via negative versus neutral emotion induction and image generation versus no image generation control groups; and Study 2 (*N* = 54), which qualitatively examined open-ended feedback. Our results are heterogeneous: both studies suggested that StoryWriter somewhat contributed to improved emotion outcomes for participants with pre-existing negative emotions, but users’ open-ended responses indicated that these outcomes may be adversely modulated by the generated images, which could undermine the therapeutic benefits of the writing task itself.

## 1. Background

Many studies have described the therapeutic benefits of expressive writing for improving resilience and managing negative emotions [1–3]. Pennebaker & Beall [4], for instance, found that writing about a traumatic event for 15 minutes a day over four days led to improved physical health six months later. Such expressive writing enables individuals to cognitively process their traumatic experiences [5], elaborate on associated negative emotions [6,7] and thereby habituate to these negative experiences [8]. Furthermore, writing therapies are easy to deploy in unsupervised settings, and may be a good option for the estimated 42% of individuals who primarily turn to the Internet for guidance on mental health issues [9]. However, research has also warned about the limited and sometimes deleterious effects of expressive writing. In a meta-analysis of 39 randomized controlled trials, Reinhold *et al.* [10] found that expressive writing exerted only limited effects in reducing depressive symptoms, and sometimes exacerbated results in unpredictable ways. In unsupervised settings, expressive writing can occasionally increase psychological distress [11,12], even to the point of causing patients to discontinue their treatment [13,14]. These conflicting findings indicate that, despite the efficacy and convenience of expressive writing for emotion regulation, care should be taken when deploying this approach in self-directed practices.

Writing can also be a form of distraction for the individual. When used appropriately, distraction tasks can alleviate negative emotions during stressful situations. In two separate experimental studies, Trask & Sigmon [15] and Nolen-Hoeksema & Morrow [16] found that participants with negative emotions (induced negative emotion, in the first case; depression in the second) who completed a distraction task reported lower levels of that negative emotion post-task than those who completed a rumination task. Distraction tasks have even been effective for short-term coping [17], and they pose little to no risk of worsening baseline emotions in depressed individuals [18]. These attributes make distraction tasks ideal for online, unsupervised settings. In this research, we specifically aim to induce positive distraction, which shifts attention away from negative stimuli [19]. To do this, we pair creative writing with representational or gentle abstract art, which has been shown to facilitate positive distraction for stress and anxiety regulation [20]. Specifically, we explore the use of text-to-image generation models to generate such images in real time based on users’ narratives.

We designed and piloted an online writing tool called StoryWriter, which pairs expressive writing and state-of-the-art, deep-learning-based text-to-image functionality, to enable ongoing positive distraction. In the application, users are tasked to write creative introductions to a fictional narrative, which in turn creates machine-generated artworks that can direct them away from negative emotions. In this paper, we describe the design, implementation and outcomes of two studies we performed to gauge the preliminary efficacy of our application. The first—an experimental study conducted with 388 users to quantify the emotional effect of the application—is recounted in §4. The second—a qualitative study with 54 remote users—was performed to provide depth and context for our other findings and is recounted in §5. After reporting the results of these studies, we discuss the benefits and limitations of our application for negative emotion downregulation.

## 2. Related work

In this section, we cover previous research related to (i) the use, benefits and disadvantages of technology-mediated writing activities and (ii) the previous application of digital technology for emotion regulation and positive distraction, as well as the potential use of artificial intelligence (AI) for similar purposes.

### 2.1. Technology-mediated writing activities

In contemporary society, writing activities are increasingly mediated through myriad technologies and carried out online. While classic expressive writing exercises used in therapeutic care are generally paper-based and mostly conducted on-site, burgeoning studies indicate that computer-based writing exercises may be equally effective while providing a more cost-effective, accessible and anonymous alternative [21–23]. Online writing activities have yielded positive user feedback and promising results in fields such as prolonged grief disorder (PGD) [7], post-traumatic stress disorders (PTSD) [24,25] and depression [26] and other mood disorders [27,28]. Thus, internet-based technologies have been applied in the field of healthcare to promote better self-monitoring [29,30]. However, researchers have also warned about the potential negative effects of technology-mediated writing tools, highlighting how unsupervised writing exercises that require users to write about distressing past events could stimulate negative emotions and reduce the exercise’s overall effectiveness [11,14].

While technology-mediated writing activities seem to provide some positive effects, they are often implemented under psycho-therapeutic protocols and in a controlled environment. We argue that, in real-life situations, individuals frequently use online resources to directly or indirectly access mental health advice. Many people cannot easily access moderated writing exercises due to financial, systemic or logistical constraints. As such, there is a need for technologies that can facilitate safe, online, emotionally regulating writing experiences.

### 2.2. Digital and artificial intelligence technologies for emotion regulation

Whereas earlier emotion regulation practices tended to rely on participants manually self-monitoring, recognizing and adjusting their emotions in response to challenging circumstances, recent advances in digital technologies have led researchers to examine how these tools might facilitate this process [31,32]. Ubiquitous technology, in particular, has shown considerable promise on this front. For instance, one study demonstrated that commercially available smart watches can be used to (i) automatically detect emotional outburst patterns for individuals with Autism Spectrum Disorders and (ii) provide them with self-regulation strategies that can reduce unregulated anger episodes [33]. Wearable devices have also been used to help calm users who are anticipating socially stressful situations (such as a public speech) [34] or undergoing exams [35].

On a different but related front, researchers have examined how tools such as a digitally mediated nature soundscape [36] and an interactive instalment featuring projected Augmented Reality and smart floor displays [37] can be used to positively distract users from stressful or anxiety-inducing situations. Immersive technologies such as virtual reality and ambient forms of digital art have been particularly effective for this purpose [20,38]. Yet, in most of these cases, the digital content within these systems needed to be manually crafted beforehand, making it difficult to tailor the content to the specific needs and preferences of each individual.

This is where AI can play a decisive role. Previous AI research on emotion regulation focused on developing algorithms to accurately detect human emotions through physiological cues like facial expressions [39,40]. This has allowed computerized systems to perceive users’ emotional states and automatically deploy various strategies to help regulate them [41,42]. Recent developments in high-performing deep learning models, particularly in the fields of image recognition and natural language processing, have made it possible to deploy AI more broadly to support emotion regulation, beyond simply detecting emotions. Examples include chat bots being developed for text-based communication platforms to enable emotional management among distributed teams [43] and deep-learning-based models being trained and used to help service employees regulate customer emotions through real-time emotional feedback [44].

Further advances in AI have led to the development of generative adversarial networks (GANs), which have made it possible for AI to create novel content such as stories, images and music [45–47]. Using such models, practitioners can generate personalized content that matches the specific characteristics of each individual, which could then be used almost immediately in emotion regulation procedures. Despite this potential, few GAN models have been applied to the issue of emotion regulation. One notable exception is an AI mirror system developed by Rajcic & McCormack [48], which uses OpenAI’s GPT-2 model to generate poetry based on users’ facial expressions and thereby provoke emotional self-reflection. To begin filling this gap, we explore the use of GAN models as a means for positive distraction. We deploy our prototype within an online writing-related exercise to ascertain whether the GAN mechanism can mitigate the issues acknowledged in §2.1.

## 3. Design and development of StoryWriter

Our design process was guided by the following objectives: (i) users should experience improved emotions after using StoryWriter for short-term coping, (ii) the mechanism must pose little to no risk of exacerbating pre-existing negative emotions, making it accessible to as many people as possible and (iii) the application’s infrastructure and image generation technology should be easy to replicate, modify and scale upwards, making it straightforward for other developers to adapt and use our work.

**Figure 1.**
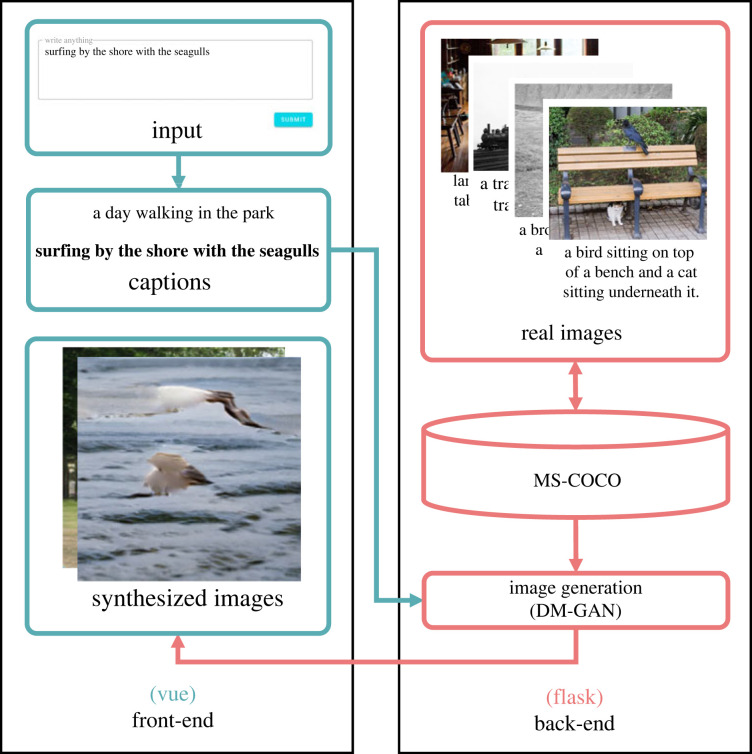
System architecture of *StoryWriter*.

To make the interface as easy to use as possible, we designed StoryWriter as a single page application (SPA). We developed it using the *Vue* framework (vue.js)^1^ for the front-end and Python’s Flask framework^2^ for the back-end in order to forward the user’s text as an input to a text-to-image generation model. Figure 1 shows the architecture of the application. Its components are described below.

### 3.1. Text-to-image generation model

Different GAN architectures offer different features that could lend themselves to text-to-image generation. For instance, StackGAN delegates image generation to multiple GAN layers, thereby increasing the resolution of each subsequent layer [49], while AttnGAN introduces an attention mechanism to match word features with subregions in the output image [46], thereby capturing fine-grained details in the generated images. We wanted a model that could solve one of the key weaknesses of a multilayered approach: that is, the way the quality of the first layer constrains the quality of the last layer [50]. Thus, we decided to use Dynamic Memory (DM)-GAN in our application, as it incorporates a memory mechanism that can refine the image contents generated in the early layers of the models, yielding higher quality images [50]. This model was trained on the MS-COCO dataset [51], a dataset comprising 123 287 images with five annotated descriptions per image. This large dataset contains a total of 886 k object instances across 80 categories.

### 3.2. User interface

**Figure 2.**
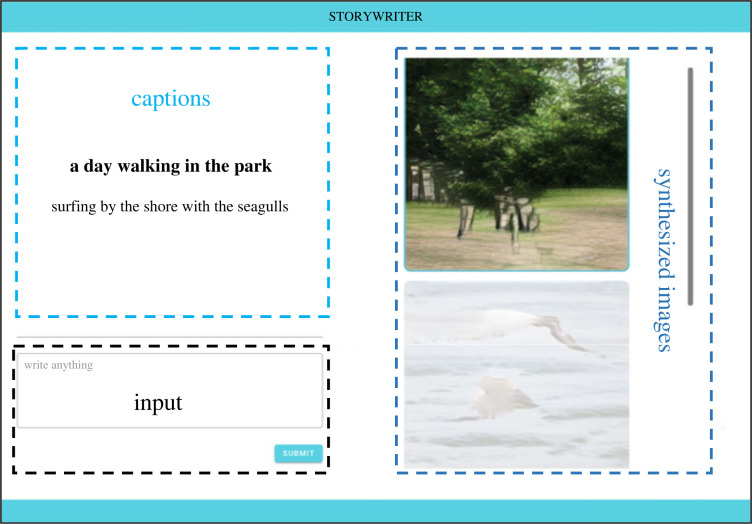
User interface of StoryWriter.

The StoryWriter interface, shown in figure 2, is divided into three main sections: Input, Synthesized Images and Captions. We describe each of these sections below.

#### 3.2.1. Input

The input area refers to the text box where users can write and submit their narratives. Users can write anything within this text area, but they are directed to submit content one sentence at a time to facilitate image generation. Once the user clicks the 'submit' button, the DM-GAN model will synthesize an image using the submitted text as an input. The application will then display the newly synthesized image in the image section, highlighting the corresponding text in the caption section.

#### 3.2.2. Synthesized images

This area displays the images generated by the DM-GAN model. Users can scroll up and select any of the previously synthesized images. Clicking or hovering over any of the images will highlight the respective user-inputted captions (i.e. the input text used to synthesize that image in the model).

#### 3.2.3. Captions

This area displays all the previous texts submitted by the user. The caption of the currently selected image is highlighted by default. As with the image area, users can scroll up and see any of the previously submitted texts.

### 3.3. Task tracking

Given the risks involved in autobiographical expressive writing and the fact that our application did not include counseling or human-based interventions, we decided not to adopt the conventional expressive writing paradigm used in offline settings, as it might prove too risky in the application’s anonymous, unsupervised, online setting. Instead, we opted to use a story-based expressive writing task that allows users to submit fictional narratives. Participants were instructed to ‘write the introduction of a short story’ and to ‘describe its setting and place while being as imaginative as possible’ (see [52]). They were also instructed to write for a set period of time (three minutes for Study 1, five minutes for Study 2).

To track the user’s interaction with the application, we added an interface mechanism to record users’ Telemetric data via timestamps and unique codes. We used SQLAlchemy to incorporate a database at the back-end of the StoryWriter application. Each user is assigned a unique seven-digit identification (ID) code, generated at their first input keystroke. The user’s first keystroke also activates a countdown timer, which, once elapsed, reveals a unique completion code that participants can input when the system is deployed as part of a survey platform (e.g. Prolific). Every time the user clicks on the ‘submit’ button to generate an image, we store the input text with a corresponding timestamp in our database. The small region in the top right of the interface displays the countdown timer and (once elapsed) the ID code used to identify the participants.

## 4. Study 1: quantitative experiment

**Figure 3.**
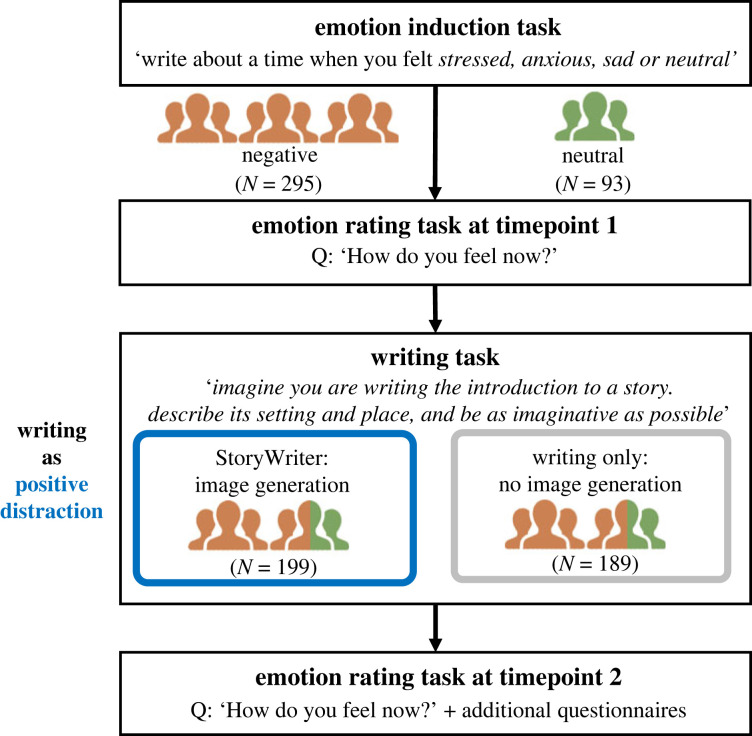
The flow of the experiment. Participants were first induced to feel a negative (versus neutral) emotion through an emotion induction task before using the StoryWriter application.

We conducted an experiment with emotion- and application-related control groups to determine StoryWriter’s efficacy in downregulating negative emotions. We hypothesized that users induced to feel negative emotions such as anger, sadness, anxiety or stress would exhibit a greater reduction of negative emotion after using StoryWriter than users assigned to the control condition. An overview of the experimental procedure can be seen in figure 3.

### 4.1. Participants

We collected data from a total of 421 participants from Prolific,^3^ an online crowd-sourcing website for psychological research. After excluding participants who failed attention checks or provided invalid responses (mismatched IDs or blank submissions), we included data from 388 participants in our study (men = 131, women = 252, other = 4, rather not say = 1; mean age = 30.1, s.d. = 8.9). Of these, 295 participants were randomly assigned to the negative emotion conditions (anxiety = 89, sadness = 99, stress = 107) and 93 participants were assigned to the neutral condition. Moreover, 199 participants were assigned to the StoryWriter (writing with image generation) condition, while 189 participants were assigned to the control (writing only) condition. Participants were compensated with GBP £2.05 for their involvement in the study. All participants held either US nationality or residence, and all but two (Spanish) reported using English as a first language. This study received approval from the Institutional Review Board of the Nara Institute of Science and Technology (2021-I-2), and participants provided informed consent before participating in the study and were allowed to withdraw from the study at any point in time. Anonymized participant data are available online at our Open Science Framework (OSF) repository https://osf.io/6cpjv/.

### 4.2. Procedure

As we were examining the efficacy of the application for users with pre-existing negative emotions, we started the study with an emotion induction task. After providing consent to participate, participants were induced to feel either negative emotions or a neutral emotion through an autobiographical recall task (see [53]) where participants recalled (and wrote about) a time they felt anxious, sad, stressed or—for the neutral condition—performed an everyday routine task. Following this, participants rated the extent to which they felt the following emotions on a seven-point Likert scale, from 1 (not at all) to 7 (extremely): happy, awe, angry, touched/moved, grateful, excited, calm, sad, anxious, stressed, generally positive and generally negative. We refer to this as Timepoint 1 (*T*1). Participants were then redirected to either the StoryWriter application or the control application (which included all the same features as the StoryWriter application except for image generation and display) and were asked to complete the task described in §3.3. They were also instructed to use the application for a minimum of three minutes. Thereafter, participants repeated the emotion rating task. We refer to this as Timepoint 2 (*T*2). Participants then evaluated the applications and completed measures of well-being (satisfaction with life scale (SWLS) [54]), psychopathological tendencies (depression, anxiety and stress scale (DASS) [55,56]) and creativity (self-perceived creativity scale (SPC) [57]). They also provided demographic information (age, gender, languages spoken, nationality). For usability and engagement, participants rated the ‘fun’ and ‘ease of use’ of the application.

### 4.3. Measurements and analyses

To evaluate the changes in emotion before and after using the applications, we created a difference score (*D*) by subtracting *T*1 ratings for each emotion category from the *T*2 scores, focusing especially on the angry, sad, anxious, stressed and generally negative items. Subjective evaluations of the application focused on emotion improvement (using this writing application helped me get into a better mood) and usability (the writing application was easy/fun to use).

We used the Telemetry infrastructure described in §3.3 to measure the character count of participants’ narratives and the time spent between the first keystroke and the last ‘submitted’ text on the application. These figures were used to evaluate the extent to which participants actively engaged with the application. Participants then copied the generated completion ID codes from the application into the survey platform, which allowed us to combine both sources of data for each user.

All analyses were conducted using 2 × 2 ANCOVAs via the Jamovi software with negative/neutral emotion induction (Emotion) and StoryWriter/control (Writing) conditions as the independent variable (IV). We also included SWLS, DASS and SPC as covariates. We used an alpha level of *p* = 0.05 for significance testing. All *p*-values reported are uncorrected. More detailed results (including non-significant findings) are available on our OSF repository (https://osf.io/6cpjv/).

### 4.4. Results and discussion

#### 4.4.1. Emotion induction

Users induced to feel negative emotions reported corresponding increases in negative emotions at *T*1. Users induced with negative emotions reported higher anger (*F*_1,192_ = 18.72, *p* < 0.001), sadness (*F*_1,182_ = 33.34, *p* < 0.001), anxiety *F*_1,142_ = 11.69, *p* < 0.001), stress (*F*_1,150_ = 15.53, *p* < 0.001) and general negativity (*F*_1,173_ = 15.28, *p* < 0.001), as well as lower calmness (*F*_1,152_ = 19.54, *p* < 0.001), than those in the neutral emotion condition. This suggests that the emotion induction task was largely effective in its intended effect, and users in the negative emotion condition began their interactions with StoryWriter from a negative emotional state.

### 4.5. Application efficacy

Of the primary variables considered in our experimental study, only anger, sadness, emotion improvement and fun yielded statistically significant overall models when designated as the dependent variable. This suggests that participants’ anger, sadness, emotion improvement and fun were meaningfully impacted by the other variables in our study. However, none of the variables had significant main effects in both the Emotion (negative versus neutral) and Writing (StoryWriter versus control) conditions. The only main effects observed were with the SPC covariate, and only among the items for emotion improvement, fun and ease of use. Namely, users with higher self-rated creativity were more likely to rate the app as effective in emotion improvement and as fun or easy to use (see table 1).

**Table 1.** Results from respective analysis of covariate (ANCOVA) models. We report the main effects of creativity (SPC) and the interaction effect between induced emotion (negative versus neutral) and task (StoryWriter versus control). A detailed list of results (including effect sizes) is available on our OSF repository.

variables	model		main effects (SPC)		interaction effects	
	*F*	*p*	*F*	*p*	*F*	*p*
*emotion*						
anger	*F*_23,364_ = 1.97	0.005**	*F*_1,364_ = 0.004	0.95	*F*_1,364_ = 0.47	0.59
sadness	*F*_23,364_ = 1.78	0.016*	*F*_1,364_ = 0.36	0.55	*F*_1,364_ = 1.32	0.25
anxiety	*F*_23,364_ = 1.01	0.448	*F*_1,364_ = 0.92	0.338	*F*_1,364_ = 2.56	0.110
stress	*F*_23,364_ = 0.49	0.070	*F*_1,364_ = 2.59	0.11	*F*_1,364_ = 0.25	0.616
negative	*F*_23,364_ = 1.24	0.208	*F*_1,364_ = 1.23	0.27	*F*_1,364_ = 0.42	0.515
positive	*F*_23,364_ = 1.34	0.139	*F*_1,364_ = 0.34	0.56	*F*_1,364_ = 0.42	0.515
*efficacy*						
emotion impr.	*F*_23,363_ = 1.99	0.005**	*F*_1,363_ = 19.79	<0.001*	*F*_1,363_ = 0.29	0.593
*usability*						
ease of use	*F*_23,363_ = 1.41	0.101	*F*_1,363_ = 4.24	0.040*	*F*_1,363_ = 0.76	0.39
fun	*F*_23,364_ = 1.99	0.005*	*F*_1,364_ = 15.97	<0.001***	*F*_1,364_ = 1.44	0.23
*engagement*						
character count	*F*_23,364_ = 1.49	0.070	*F*_1,364_ = 3.15	0.077	*F*_1,364_ = 0.91	0.339
time spent	*F*_23,364_ = 0.41	0.993	*F*_1,364_ = 0.06	0.80	*F*_1,364_ = 0.39	0.530

*p < 0.05, **p < 0.01 and ***p < 0.001.

**Figure 4.**
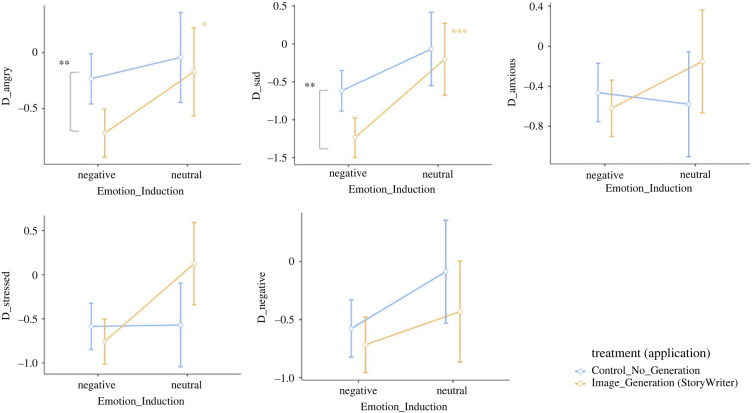
The results of the StoryWriter/control application for regulating negative emotion. ‘*’ indicates *p* < 0.05, ‘**’ indicates *p* < 0.01 and ‘***’ indicates *p* < 0.001.

**Figure 5.**
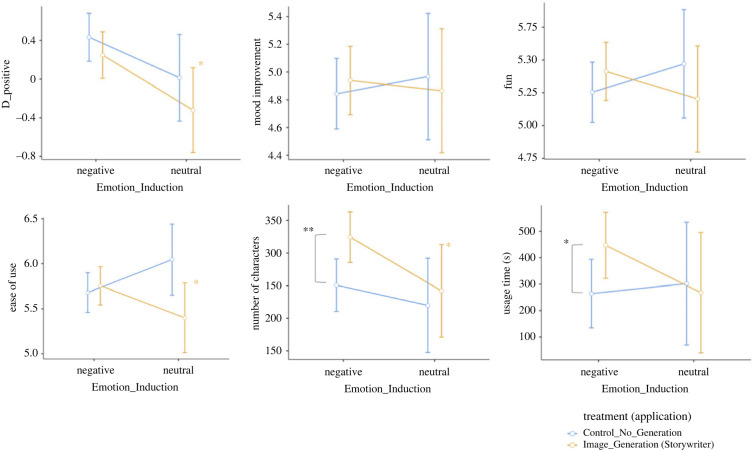
The results of the StoryWriter/control applications for emotion improvement, usability, engagement and Telemetry. ‘*’ indicates *p* < 0.05 and ‘**’ indicates *p* < 0.01.

**Figure 6.**
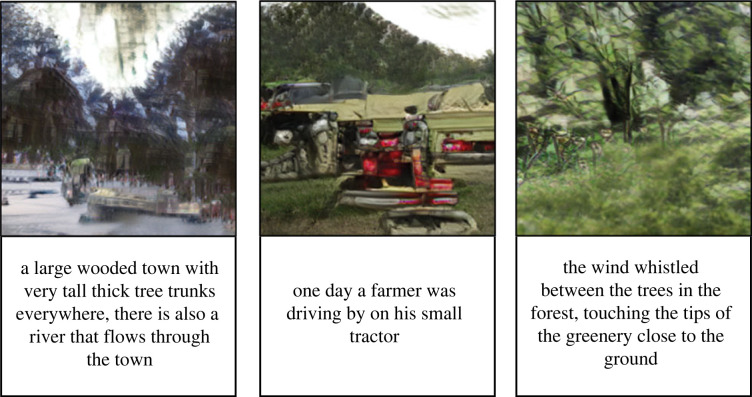
Examples of written narratives and the respective images synthesized by the text-to-image DM-GAN model.

Figure 4 shows the results of the StoryWriter and control conditions for our hypothesized effect (regulating negative emotions). Figure 5 shows the results for emotion improvement, usability, engagement and Telemetric data. Figure 6 shows some examples of submitted narratives with their respective output images.

Results of planned *post hoc* comparisons are shown in table 2. Significant differences were observed between variables across conditions. Anger, sadness and character count yielded differences between both sets of conditions: users assigned to StoryWriter exhibited significantly reduced anger and sadness and increased character counts when induced with negative rather than neutral emotions, while users assigned to the negative emotion condition exhibited similar outcomes when using StoryWriter as opposed to the control application. Some variables, such as stress, general positive affect, ease of use and time spent, exhibited significant differences in only one set of study conditions. For instance, users assigned to StoryWriter exhibited significantly reduced stress and significantly increased general positive affect when induced with negative rather than neutral emotions, but there were no significant differences for users assigned to the negative emotion condition when they used StoryWriter compared to the control application. On the other hand, users assigned to the negative emotion condition spent significantly more time writing when they used StoryWriter rather than the control application, but there were no significant differences in writing time for StoryWriter users assigned to the negative versus neutral emotion condition. Participants in the control condition generally found the application easier to use than those in the StoryWriter condition (*F*_1,363_ = 5.50, *p* = 0.020), which is unsurprising given the relative difficulty of writing for image generation purposes. Finally, variables like anxiety, general negative affect, emotion improvement and fun yielded no significant differences between either set of study conditions. Moreover, none of the variables had significant interaction effects between the Emotion and Writing conditions.

**Table 2.** Table of planned *post hoc* pairwise comparisons. Effect sizes (Cohen’s *d*) were reported only for significant effects.

variables	StoryWriter								negative induction							
	negative		neutral						StoryWriter		control					
	*M*	s.e.	*M*	s.e.	*t*	d.f.	*p*	*d*	*M*	s.e.	*M*	s.e.	*t*	d.f.	*p*	*d*
*emotion*																
anger	−0.72	0.11	−0.17	0.20	−2.40	364	0.017*	−0.40	−0.72	0.11	−0.23	0.11	3.07	364	0.002**	0.36
sadness	−1.24	0.13	−0.20	0.24	−3.76	364	<0.001***	−0.63	−1.24	0.13	−0.62	0.14	3.24	364	0.001**	0.38
anxiety	−0.61	0.14	−0.15	0.26	−1.58	364	0.116	—	−0.61	0.14	−0.46	0.15	0.76	364	0.445	—
stress	−0.76	0.13	−0.13	0.24	−3.27	364	0.001**	−0.55	−0.76	0.13	−0.59	0.13	0.92	364	0.359	—
neg. affect	−0.72	0.12	−0.43	0.22	−1.14	364	0.257	—	−0.72	0.12	−0.58	0.13	0.80	364	0.423	—
pos. affect	0.25	0.12	−0.32	0.22	2.24	364	0.026*	0.38	0.25	0.12	0.43	0.13	1.05	364	0.294	—
*efficacy*																
emotion impr.	4.94	0.13	4.87	0.223	0.28	364	0.776	—	4.94	0.13	4.84	0.13	−0.53	364	0.595	—
*usability*																
ease of use	5.76	0.11	5.40	0.20	1.58	363	0.115	—	5.76	0.11	5.68	0.11	−0.48	363	0.631	—
Fun	5.41	0.11	5.20	0.21	0.89	364	0.372	—	5.41	0.11	5.25	0.12	−0.98	364	0.328	—
*engagement*																
cha. count	324	19.8	242	36.0	−2.04	364	0.046*	0.34	324	19.8	251	20.5	−2.56	364	0.010*	−0.30
time spent	447	63.6	268	115.9	1.36	364	0.176	—	447	63.6	264	65.8	−2.00	364	0.046*	−0.23

*p < 0.05, **p < 0.01 and ***p < 0.001.

In short, while there is some evidence that (i) StoryWriter allowed users to positively distract themselves from negative emotions and (ii) self-reported creativity had an impact on users’ engagement with the application, we do not have sufficient evidence to determine the overall efficacy of StoryWriter in achieving its intended effect. Moreover, we examined individuals with induced negative emotional states, which differs from our initial objective of assessing positive distraction through creative writing for depressed individuals. Given the complexity of these unanswered questions, we decided to run a qualitative user study focusing more on the mechanisms involved in positive distraction among depressive StoryWriter users.

## 5. Study 2: qualitative user study

In Study 2, we aimed to examine how users in our target user-base (users with depression) perceived the experience of using StoryWriter. Though we collected stories and images from the experimental study, we were eager to solicit users’ appraisals of the images and obtain more open-ended feedback about their emotions after using the application. Due to operational constraints and the ongoing impact of COVID-19, we chose to administer this qualitative component remotely as a long-form questionnaire to build a foundational understanding of user impressions that could inform future user studies involving text-to-image therapeutic writing applications.

### 5.1. Participants

We first used Prolific to recruit two sets of participants to a prescreening survey, which included the DASS, SPC and SWLS inventories from Study 1, alongside demographic questions and questions about participants’ creative pastimes (both writing and other artforms), mental health diagnoses and COVID and holiday-related experiences (the study was completed between December 2021 and January 2022). This would provide context for their current emotional baseline and writing approach. The first participant set targeted a general population above the age of 20 who are located in the US (following Study 1’s inclusion criteria), while the second set included such users with past mental health conditions (as a filter applied through Prolific). We used a stratified sampling strategy to ensure that results reflect diverse perspectives—especially given the prospective therapeutic use of our application—while also including enough neutral-range participants to contextualize findings from Study 1.

From the 314 individuals who engaged with our prescreening survey (233 from the mental health-specific group, 81 from the general population), we received 302 responses, 271 of which were complete and usable. After excluding one entry that did not meet the inclusion criteria, we ranked the remaining entries based on their response quality (length and depth), DASS scores, SPC scores and diversity, prioritizing greater engagement with the survey and more stratified DASS scores (highest and lowest) while taking care to include some participants with the least represented creativity and demographic information in the sample (for instance, low SPC, high income, etc.). Ultimately, we invited 100 of these individuals to participate in the main qualitative survey—50 each from the high and low DASS groups (glossed as the ‘DASS’ and ‘Neutral’ groups). Of these, 65 responded, yielding 62 submitted surveys, 54 of which were complete and usable (31 DASS, 23 Neutral). Thus, our analysis includes 54 participants (30 women, 19 men, 5 non-binary; mean age = 33.7, s.d. = 11.7) who are either US citizens or residents and who, with one exception (Chinese), speak English as their dominant language. Participants in the prescreening survey were compensated with GBP £0.84, while participants in the main survey were compensated with GBP £3.00. This study received approval from the Institutional Review Board of the Nara Institute of Science and Technology (2021-I-2), and participants provided informed consent before participating in the study, and were allowed to withdraw from the study at any point in time.

### 5.2. Procedure

All participants in the main survey engaged with the StoryWriter application (the control application from Study 1 was not used in Study 2). We devised two versions of the same survey: one with an induction task that invited respondents to reflect on a recent sadness-, anxiety- or stress-inducing experience (following Study 1), and one without this task. This was to examine differences between baseline (typical) and escalated (atypical) negative emotions. To account for extraneous factors and ascertain baseline emotions, each survey began with an open-ended question inviting participants to talk about their day and current feelings, as well as a multi-select question where participants could record common external factors contributing to their current mindset (e.g. tiredness or physical discomfort, preoccupation with life events, etc.). We did not include structured emotion checks because the objective was not to use this information for empirical analysis, and because we did not want to cause participants to draw preemptive conclusions about the purpose of our study (which could affect the way they answered later questions).

We directed DASS and Neutral participants into each of the two surveys (glossed here as ‘ES’, for the emotion induction survey, and ‘NS’, for the naturalistic (no-induction) survey), taking particular care to allocate enough DASS users to the ES survey to provide insight into prospective application use cases; 19 DASS and 10 Neutral participants completed the ES survey, while 12 DASS and 13 Neutral participants completed the NS survey. After providing consent to participate in the study, participants completed the brief reflection, the priming task (where applicable) and then immediately proceeded to sample the StoryWriter tool with the same task used in the experimental study (§3.3). For this study, we adjusted the timer from three to five minutes in order to encourage slightly longer stories, and we adjusted the instruction text with updated study information, but preserved all other features.

Following the StoryWriter segment, participants were invited to reflect on their writing process, including the decisions that went into their particular story and the overall ease or difficulty of writing. Thereafter, participants were asked to elaborate on their current emotional state and to rank the factors influencing their emotions (those specified at the beginning of the survey alongside StoryWriter components: interface, images and writing process). Upon completing this portion, participants proceeded to answer a set of user experience questions (what did you like and dislike about the application? what did you think of the application’s layout?), questions about the images’ suitability and effect on the writing process, and questions about their satisfaction with their story and appraisal of the StoryWriter application.

### 5.3. Analyses

We used NVivo to process and code the survey responses and the accompanying stories and images. One researcher undertook this portion of the project, deploying an analysis approach that is best described as Template Analysis: an iterative but systematic mode of thematic analysis involving the initial development and subsequent adjustment of a coding ‘template’ [58]. This means that codes were developed both deductively (from pre-defined themes in Study 1) and inductively (based on new patterns observed in the data; [58, p. 203]). *A priori* themes included positive versus negative versus neutral distraction, emotion change and valence, and specific emotion groups (anger, sadness, anxiety, stress), and they were progressively tailored to shed light on the following questions: (i) what do distraction and engagement look like in practice? (ii) if there is distraction, what precisely is causing it? (iii) what makes distraction positive (versus neutral or negative)? Furthermore, as per Template Analysis, codes were arranged both hierarchically (within broader themes) and laterally (based on ‘integrative’ resonances across clusters; [58, p. 204]). Our hierarchical arrangements tended to specify domains of data, or topic groups informed by our survey questions (our final template is organized by survey phases, like Pre-Study, Study, Stories and Images, and by application features: images, interface, writing), whereas our lateral arrangements specified emotions that appeared at various stages of the study. We determined this scope to be sufficient for describing individuals’ views and experiences over the course of their interaction with the tool, and for the purpose of contextualizing the findings of Study 1.

We generally favoured semantic (rather than latent) meanings, or codes that described participants’ wording/express orientations, though we did interpret latencies in some behaviours—for example, negative distraction in instances where participants deliberately avoided looking at the StoryWriter images. These latent meanings are not intended as absolute standards for how the material should be interpreted, but as one of many possible interpretations that can enrich our understanding of the observed phenomena (e.g. distraction, engagement, emotion downregulation, etc.). Indeed, we took care during the analysis to identify alternate ways in which the target phenomena were being described—for instance, distraction occurring relative to the writing process (as a negative, unwelcome detour) rather than to negative emotions (as a positive intervention, as defined in Study 1). Thus, we would say that we performed our analysis from a ‘contextual constructivist’ position [59], which ‘assumes that there are always multiple interpretations to be made of any phenomenon, and that these depend upon the position of the researcher and the specific social context of the research’ [58, p. 205]. This position is not typically amenable to quantitative reliability measures like inter-rater reliability, given that ‘all accounts, whether those of participants or of researchers, are understood to be imbued with subjectivity and therefore not *prima facie* invalidated by conflicting with alternative perspectives' [59, pp. 9–10]. Rather, we pursued consistency and comprehensiveness by (i) designing our survey to examine given concepts from multiple vantage points (for instance, through open-ended questions as well as scales and ranking questions), providing a degree of triangulation that will become evident in our results and discussion; (ii) recruiting new participants until we were satisfied with the coverage of topics and (iii) reviewing our codes and transcripts several times, as per typical Template Analysis procedure, and redeploying/standardizing the template until it was able to accommodate all parts of the participants’ responses to the survey questions.

Emotional states were coded at four points: baseline (the first emotion check-in question), induction (prompt that asked participants to reflect upon and recount a recent negative event), story (based on the emotions conveyed by characters or projected onto the story environment) and post-study (emotional check-in question). We began by extracting the affective language used by the participants (e.g.*irritated*), then grouped these together into related sentiment groups (e.g. *irritated*, *annoyed*, *bothered*), which became part of our larger, *a priori* emotion themes (namely, anger-adjacent, anxiety/stress-adjacent and sadness-adjacent). We distinguished stress from anxiety based on the degree to which the sentiment could be interpreted as situational (instigated by circumstances) versus dispositional (endemic, habitual). Changes in emotional states were determined primarily based on changes in the number of items coded to each emotion code, and corroborated by examining emotion change in terms of the presence of new affective language categories or absence of previous ones.

### 5.4. Results and discussion

Our overarching assumption was that GAN-generated images could help to moderate users’ negative emotions during online writing activities by providing positive distraction. Additionally, we hypothesized that participants induced with negative emotions would achieve better emotional outcomes than neutral participants. Below we discuss these results in light of the qualitative findings.

#### 5.4.1. Negative emotion downregulation

According to the results of Study 1, participants with negative emotions who used StoryWriter exhibited small but significant reductions in anger and sadness, and some reduction in stress, particularly when compared to users who either did not use the StoryWriter application or users who were not induced with negative emotions prior to using the application.

**Figure 7.**
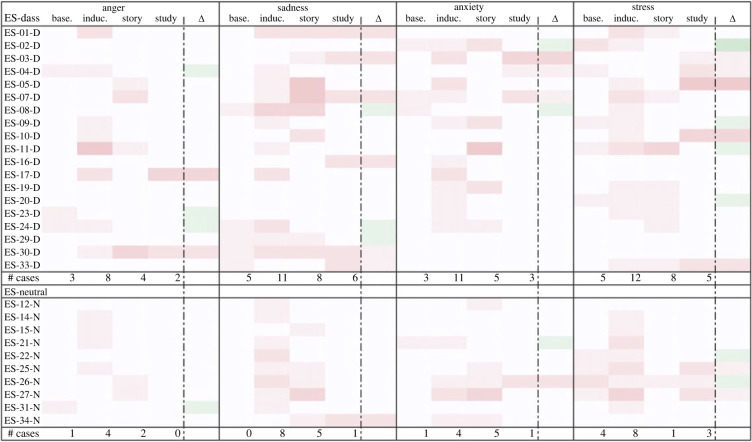
Negative emotional trajectory of participants in the ES survey. Leftmost column lists the participants; centre columns indicate instances of the given emotion at each time point (greater saturation represents more instances); rightmost column indicates change in the given emotion between baseline and post-study time points (more green represents lower presence of given negative emotion, more red represents greater presence of given negative emotion). # Cases = number of participants who reported given emotion at each time point.

**Figure 8.**
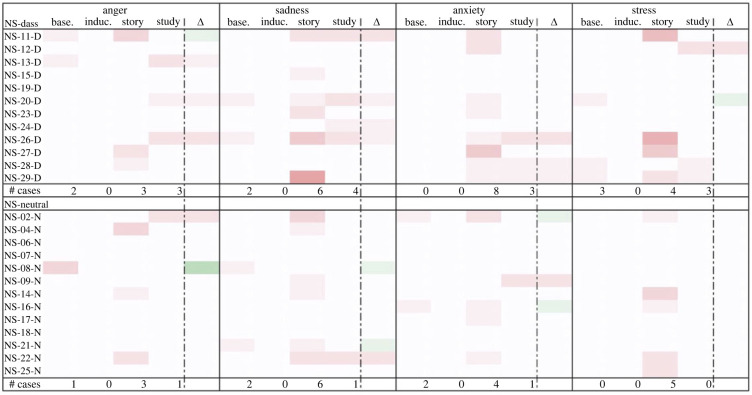
Negative emotional trajectory of participants in the NS survey. Leftmost column lists the participants; centre columns indicate instances of the given emotion at each time point (greater saturation represents more instances); rightmost column indicates change in the given emotion between baseline and post-study time points (more green represents lower presence of given negative emotion, more red represents greater presence of given negative emotion). # Cases = number of participants who reported given emotion at each time point.

**Figure 9.**
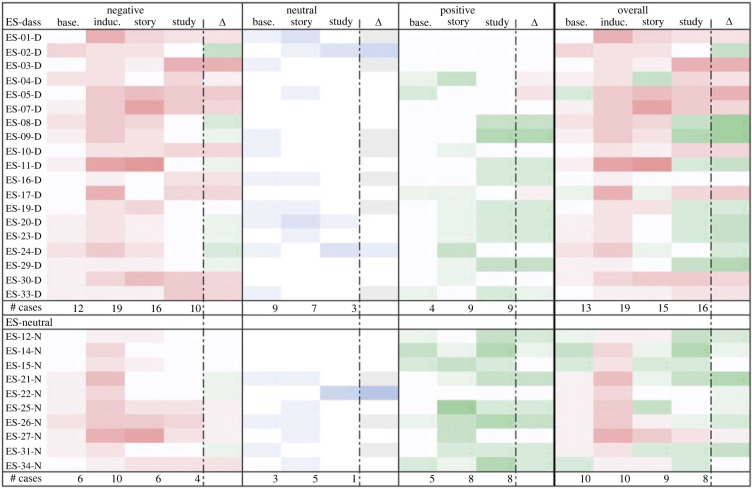
Total emotional trajectory of participants in the ES survey. Leftmost column lists the participants; centre columns indicate the sum of given emotions at each time point (positive emotions as positive values, negative emotions as negative values, neutral emotions as zero value); rightmost column indicates change in total emotional valence between baseline and post-study time points (more green represents more positive emotion change, more red represents more negative emotion change). # Cases = number of participants who reported a given emotion at each time point.

**Figure 10.**
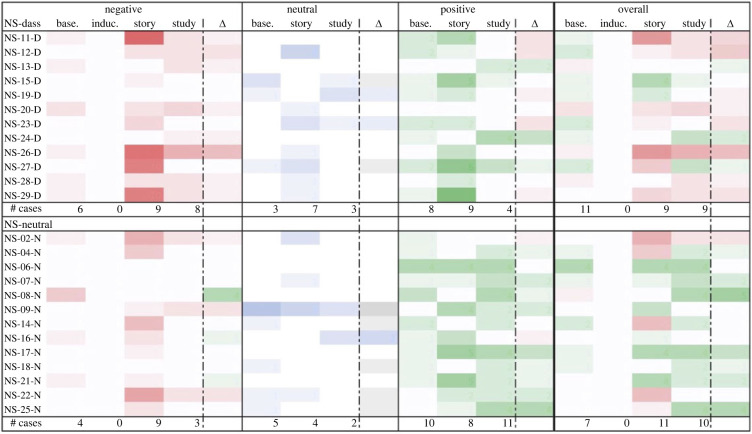
Total emotional trajectory of participants in the NS survey. Leftmost column lists the participants; centre columns indicate the sum of given emotions at each time point (positive emotions as positive values, negative emotions as negative values, neutral emotions as zero value); rightmost column indicates change in total emotional valence between baseline and post-study time points (more green represents more positive emotion change, more red represents more negative emotion change). # Cases = number of participants who reported a given emotion at each time point.

Similarly, though Study 2’s qualitative results provided only limited evidence for negative emotion downregulation due to the small sample size, it did appear that more negative baseline emotions corresponded somewhat with better emotion outcomes post-study (see figures 7–10). Induced emotional escalation, however, did not correspond much with emotion outcomes.

**Figure 11.**
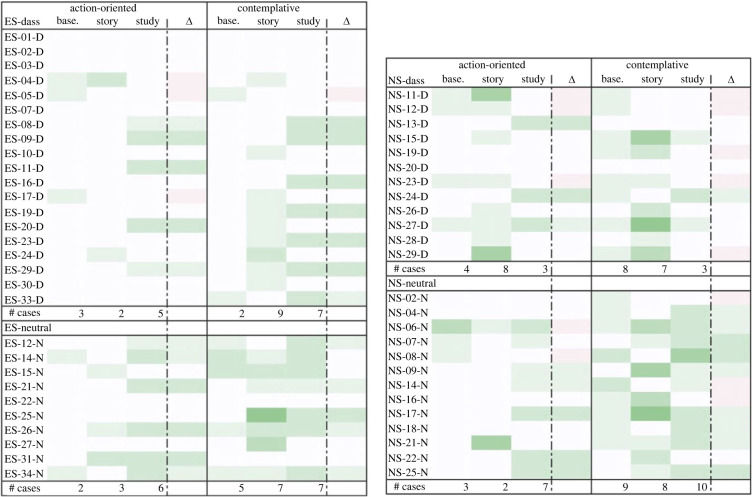
Positive emotional trajectory of participants. Leftmost column lists the participants; centre columns indicate instances of the given emotion at each time point (greater saturation represents more instances); rightmost column indicates change in the given emotion between baseline and post-study time points (more green represents greater presence of given positive emotion, more red represents lower presence of given positive emotion). # Cases = number of participants who reported given emotion at each time point.

Over the course of thematic analysis, we assembled positive emotions into two groups (or themes), which we glossed as ‘action-oriented’ and ‘contemplative’. Action-oriented positive emotions included adjectives like capable, competent, motivated, excited, curious, stimulated and so on—all words that seemed to suggest a subsequent action (capable of…, motivated to, curious to [see]…, etc.). Contemplative positive emotions included adjectives like comfortable, content, grateful, happy, hopeful, calm, etc.—words that seemed to suggest a kind of appreciative stasis. The distinction partly resembles the situational versus dispositional divide used to distinguish stress from anxiety in user responses. Interestingly, there was a notable increase in action-oriented positive words over the course of the study, and in the number of participants who used such words (12 at baseline, 21 post-study). Contemplative positive emotions appeared more consistently, at least in the overarching group (there was a sharper increase among DASS participants in the ES survey), with 24 participants reporting contemplative emotions at baseline compared to 27 post-study (see figure 11).

These findings complement the results of Study 1, which showed that negative pre-existing emotions yielded generally more positive emotion outcomes, but with differential effects for different emotions. The stronger results for anger and sadness in Study 1 (as compared to anxiety and stress) may be due to the approach (versus avoidant) nature of these negative emotions [60]. Approach orientation (or action tendencies) describe the motivational system that drives individuals towards a certain desired goal or target. This is opposed to avoidant orientation, which describes the motivational system that drives users to withdraw from a target [61]. Sadness and anger—and, arguably, the specified action-oriented positive emotions—are associated with approach orientations: anger (and motivation, curiosity, competency, etc.) directly motivates individuals to approach that goal, and sadness arises from the presence of an unattainable goal [62]. While anger and sadness represent opposing ends of the approach–motivational spectrum, they nevertheless are mutually defined by their ‘approach’ orientation towards a stimulus. Thus, anger and sadness may have been more effectively mitigated in Study 1—and action-oriented positive emotions may have been more consistently bolstered in Study 2—due to our external stimulus (StoryWriter), which distracts users by redirecting them towards a goal-oriented writing task. This congruence between emotion and task could drive an orientation-matching effect [62], thereby reducing the intensity of anger and sadness. By contrast, anxiety is avoidance-oriented, stress shows differing patterns and contemplative emotions were defined by their relative stasis, so the goal-oriented distraction task employed here may not be as effective in downregulating anxiety and stress or upregulating contemplative positive emotions.

One question that remains involves the role of affect in the stories, given that our tool is designed to downregulate negative emotions that come up in the process of writing. Hypothetically, participants who used more negative words in their story should have gleaned more benefit from the image distraction. However, there was not much correspondence between negative story affect and post-study emotion outcomes, apart from outlier cases with very negative story affect, which seemed to correspond somewhat with worse emotion outcomes.

#### 5.4.2. Engagement enhancement

Based on the Telemetry results of Study 1, users induced to feel negative emotions appeared to engage more with the writing task, writing longer passages and spending more time on the application. Previous research indicated that greater engagement yields better response quality [63,64], hence our findings likely suggested that StoryWriter could support therapeutic outcomes, as meaningful engagement and thoughtful outputs are crucial to the efficacy of writing exercises.

One potential concern is that engagement is not a singular and homogeneous construct—there may be different registers of engagement that cannot be detected through time spent and character counts alone. For instance, four participants in Study 2 described their engagement with the application as a form of entertainment—like a game, a novelty or something fun to explore with friends—but not as a writing tool that they would apply to serious purposes. Moreover, six of the 15 respondents who indicated that they would not use the application for their own writing were quite prolific in their writing outputs, writing above the average character count of 551.39 and, in three cases, above 1000.

More importantly, it is not immediately clear from time and character count figures *what* participants are engaging with when they use the application. Indeed, 35 of the 54 participants (64.82%) exhibited some kind of lack of engagement with the StoryWriter images. In some cases, this may have been due to a technical problem, like device compatibility issues (*n* = 5), in others to users’ poor appraisal of the images themselves (*n* = 11), either as blurry and indecipherable or as unrelated to the story at hand. In most cases (n=30,55.56%), participants indicated that the images had no effect on their writing process. By contrast, 14 (25.93%) participants reportedly engaged with some other aspect of the interface—specifically, the way the text formatting and simplicity of the tool helped them to focus on the writing process itself. One commented, ‘It helped me take the writing process one sentence at a time, which isn’t my usual style but was good for therapy purposes’ (ES-31-N, woman, 26 years old). Another stated, ‘I occasionally write short stories and this would be a great application to use. I write my short stories usually in MS Word and that application can be very distracting. It offers [way] too many choices and most of them I don’t need’ (NS-20-D, man, 35 years old).

In the qualitative study, we used two tactics/constructs to approximate the extent and object of users’ engagement with the application: ease of writing and effect ranking. After participants had sampled the tool, we asked them, ‘How easy or difficult was it to continue writing? Were there any points where you wanted to stop writing or using the application?’ Though 50.00% (*n* = 27) of the participants found it easy to write, there was an even split between those who were motivated to keep writing (n=12,22.22%) and those who wished to stop writing (n=12,22.22%), either because of the submission pacing (i.e. the need to press enter/submit after each written sentence; *n* = 4), the images themselves (*n* = 3), or some other factor (*n* = 5). One stated, ‘The images were very distracting. When the first image appeared, I really wanted to stop using the application all together. It [got] better the further I went, though’ (ES-20-D, woman, 22 years old).

After reflecting on the writing process, participants reviewed their current emotions and attributed them to either study-related or previously registered factors (e.g. tiredness, physical discomfort, preoccupation with life events, etc.). Of the 54 total participants, 22 (40.74%) attributed their emotions to the writing process first; 19 (35.19%) ranked non-study-related factors first; five (9.26%) ranked the StoryWriter interface first and four (7.41%) ranked the StoryWriter images first (four indicated that none of the factors influenced their current emotion). Thus, the writing process was the only StoryWriter component deemed more effective (or engaging), on average, than other circumstances outside the app itself. In this light, any engagement outcomes noted in the experimental study could derive from the process of imaginative writing itself, which would explain the lack of significant differences in many emotion reduction outcomes between the StoryWriter and control groups for Study 1. The effect rankings and user commentary—which often framed the interface and images as relative/contingent to the user stories—indicate that the StoryWriter interface and images may only support negative emotion downregulation insofar as they support or mediate this writing process, rather than yielding distinct downregulating effects in and of themselves.

#### 5.4.3. Positive distraction

In Study 1, engagement was used as a proxy for distraction, with greater engagement indicating that users were more distracted by the mechanism. Additionally, positive distraction was defined as distraction that yielded positive emotional outcomes. Given these premises, the Telemetry results from Study 1 supported the conclusion that StoryWriter achieves some degree of negative emotion downregulation (for anger and sadness, and primarily among negatively induced participants) through positive distraction. This would add AI-based interventions to the list of viable positive distraction tactics available for online settings (alongside digital art instalments [37,38] or active imagination tasks in supervised therapeutic activities [15,16]).

In Study 2, we ascertained distraction through participants’ responses to questions such as ‘Why do you feel this way?’ (following the emotion check-in) and ‘How did the images affect your writing process, and what relationship did they have to your story?’ We could also observe the extent to which study elements (like the interface, images or writing process) superseded circumstantial factors in the aforementioned effect ranking. Moreover, we evaluated whether any given distraction was positive, neutral or negative based on users’ description of how certain features helped (positive) or hurt (negative) their writing process.

Of the 54 total participants, 27 (50.00%) explicitly stated that some study-related element affected their emotions. For instance, one participant who wrote a story based on recent events said, ‘I think I feel this way because I’d been disappointed about the weather, and writing about it helped me process that (and ’strike back’ at the snow)’ (NS-08-N, woman, 42 years old). Another explained their more negative sentiments, saying, ‘That’s what the images made me feel like. The sadness is from what I was writing and the anxiousness is from the images and the recounting I had to do beforehand’ (ES-03-D, woman, 20 years old). Fourteen of the 27 (or 51.85% of those who confirmed that some study-related element affected their emotions) indicated that they had been positively affected, 13 due to the writing process (ex., ‘I think the writing process was therapeutic and helped take my mind off my current troubles’; ES-31-N, woman, 26 years old), and one due to the app’s overall distraction (‘I have at minimum…been effectively distracted from what I was doing and how I was feeling previously’; ES-11-D, woman, 41 years old). Four of the 27 (14.82%) were ambivalently affected, two due to the writing task or writing subject (ex., ‘I feel [a bit sad but also·· ·hopeful] because I was thinking of issues that are very complex but with science there is hope for these issues being more understood and improved upon every day’; ES-34-N, woman, 39 years old), one due to the overall app experience (‘I don’t know why I feel [a bit disassociated], other than it’s the first time I’ve had to myself all day and this exercise was a little unusual’; NS-16-N, woman, 45 years old), and one due to the survey (annoyed because ‘The story portion was fun and imaginative but now we’re on survey questions which don’t really require imagination’; NS-13-D, woman, 50 years old). Nine participants (33.33%) reported that they were negatively affected, four because of the images (ex., disturbed because ‘It seemed to stack a lot of body parts or like parts of human bodies with animal bodies together and certain things just looked like chaotic’; NS-12-D, woman, 52 years old), two because of the writing process (ex., ‘I always feel [depressed] when I start thinking. And to write a story, I need to think’; ES-07-D, woman, 22 years old), one because of the interface (lost, frustrated because ‘I wish I could go back and change sentences to add more detail I’d forgotten about’; ES-17-D, man, 23 years old), one because the survey reset (‘It’s taken a toll on my focus and spirits to have to spend so much time redoing my work here’; NS-02-N, man, 31 years old), and one because of the priming task (‘I feel like this because yesterday’s events were very unpleasant and sad. It also reminded me that my friends didn’t even ask if I had a good day yesterday’; ES-01-D, woman, 37 years old).

Overall, participants who explicitly reported more positive emotion changes tended to attribute this to the writing process, rather than to the application or images; those who explicitly attributed their emotions to the images tended to describe negative emotion change. This is somewhat reinforced by the effect ranking, where those who listed the writing process among the top three factors for their current emotion tended to report positive emotions (19 positive versus 10 negative, 18 other), while those who listed the interface or image within the top three tended to report more neutral or ambivalent emotions (interface: 20 neutral versus 11 positive, 5 negative; images: 24 neutral versus 11 positive, 5 negative). Indeed, only one of the participants who ranked the StoryWriter images above extraneous factors and the writing process itself (*n* = 5) exhibited positive emotional change (one exhibited neutral emotion both before and after, and three reported worse emotions after the exercise because they found the images unsettling). The effect ranking also indicated that study elements superseded circumstantial factors for 23 participants (42.59%), and that four of the 35 individuals who had previously reported a non-study-related emotion influence ceased to consider it an influence after using the tool.

It is possible that the images/interface had a subconscious effect on participants, even if they did not report image/interface effects. For instance, many participants did acknowledge that the images successfully redirected their attention (*n* = 28, 51.85%). Twelve of the 28 individuals (or 42.86%) thought that the images facilitated their writing. For example, participants used them to generate ideas (ex. ‘I mostly used them when I was stuck. I would pause and then kind of let my thoughts wander and then look over at the images and see if I could generate anything based on them’; NS-11-D, man, 34 years old), visualize their stories (‘They seemed to generate images of what my characters looked like, and I took that inspiration to describe them’; ES-25-N, non-binary, 24 years old), or adjust their tone (‘I think they prompted me to be a bit darker and more fantastical than the simple romantic morning I initially planned to write about’; NS-27-D, man, 22 years old). Eight participants (28.57%) said that the images rendered no effect on their writing or emotions, while another eight reported that the images undermined the writing process to the extent that they avoided looking at/engaging with the images at all. For example, one participant stated, ‘I enjoyed the thinking, planning and writing, but didn’t like the pictures that came up next to each sentence. They threw my thoughts off balance and felt a bit creepy’ (NS-06-N, woman, 48 years old). Another specified that their writing process was ‘hindered…with the pictures that made no sense’ (NS-13-D, woman, 50 years old).

Thus, while it is clear that StoryWriter offers a good deal of distraction, it is unclear to what extent we can label the distraction as positive. This is especially evident because, among the 12 who reported positive image effects for writing, only four reported improved emotions thereafter (five reported worsened emotions). Only one of these 12 participants explicitly linked their emotion changes to the images themselves, and only five ranked images above extraneous factors in terms of their effect on their emotions.

#### 5.4.4. Users’ evaluations of the StoryWriter features

In Study 1, participants evaluated the efficacy, usability and experience of using StoryWriter, ultimately determining that it was slightly more fun, harder to use and somewhat more efficacious for emotion improvement than the control. In Study 2, we provided participants with opportunities to divulge their impressions of the images, interface and writing results in a more open-ended way, alongside more structured rankings for ease of use and image suitability.

*Interface*. Of the 44 participants who provided descriptions of the interface, 40 (90.91%) used positive words to describe the layout, usability or application overall. In terms of layout, participants described the app as *functional* (*n* = 6), *visually appealing* (*n* = 3), and especially *simple*, *clean* or *minimal* (*n* = 18). On the usability side, participants described the app as *fun*, *entertaining* or *funny* (*n* = 5), *helpful,* *useful* or *convenient* (*n* = 3), *intriguing* (*n* = 2) and especially *easy to use* or *easy to read* (*n* = 17). This was more or less corroborated by the numeric ease of use score participants assigned through a sliding scale, with zero indicating extreme ease and 100 extreme difficulty (the average for all participants was 25.98, s.d. = 22.30). Positive descriptions of the app as a whole included adjectives like *nice* (*n* = 7), *novel* (*n* = 1) and *quick* (*n* = 1). Meanwhile, 10 of the 44 specified participants (22.73%) used neutral words to describe the app, such as *fine* or *okay*, while four (9.10%) used negative descriptors such as *awkward*, *confusing* or *plain*. Despite the predominantly positive appraisal, 41 (75.93%) of the 54 total participants frequently acknowledged specific issues with the interface. Foremost among these was bad pacing, or the way that submitting sentences one at a time slowed down the writing process (*n* = 27). Other issues included the lack of some word processing or customization features (*n* = 16), including the ability to tailor the AI itself; layout grievances, such as the location of the instructions, the size of the text box or the relative positions of the text input and image output (*n* = 12); glitch and compatibility issues, namely with mobile phones (*n* = 11) and punctuation and formatting issues, such as the omission of full stops (*n* = 9).

*Images*. Of the 31 participants who deployed adjectives to describe the StoryWriter images, 22 (70.97%) included negative words like *scary*, *unsettling* or *creepy* (*n* = 15), *confusing*, *chaotic* or *incoherent* (*n* = 6), *stressful* (*n* = 1) and *boring* (*n* = 1). Twelve (38.71%) included neutral descriptors like *abstract* (*n* = 6), *curious,* *odd* or *peculiar* (*n* = 6), *okay* (*n* = 2) or *psychedelic* (*n* = 1). Thirteen (41.94%) included positive descriptors like *interesting* or *imaginative* (*n* = 12), *funny* (*n* = 2) and *relaxing* (*n* = 1). Of the 54 total participants, 38 individuals (70.37%) reported specific issues with the images. These participants took particular issue with the irrelevance of the images for their stories (*n* = 28), and with the quality of the images as a whole (*n* = 24). Some even commented on the limited scope of the images (*n* = 6), in that there was little variation between images and no opportunity to select between image options. One suggested that images be excluded from the application altogether. Nonetheless, 46.30% of all participants (*n* = 25) liked the concept of automatic image generation in theory, describing it as *interesting*, *fun* or *novel*, and some did glean actual enjoyment from the application and/or confirm that some of the images matched the tone of their story (n=7,12.96%). Overall, the average suitability rating (indicated on a sliding scale) suggested that only 26.63% of images were suitable for the writing task.

*Writing*. After completing most of the survey, participants were asked to disclose their satisfaction with their story as a way of evaluating the writing process. Roughly half (n=28,51.85%) of all participants reported that they were satisfied with their story, with some (n=5,9.26%) indicating that there were aspects of their story that they would like to explore further. Fifteen individuals (27.78%) were not satisfied with their story, and 11 (20.37%) felt somewhat neutral about their story. In terms of issues or suggestions, 16 participants (29.63%) imagined improvements to the writing prompt and instructions, or to the writing tool itself so as to enable more intentional, guided writing. One participant explained, ‘I would like if there was a new prompt to each submission. I keep seeing the same prompt (write the introduction of a short story) at the instructions section but I’ve already wrote a body and an ending’ (ES-07-D, woman, 22 years old). Another suggested, ‘Have a box at the side that threw out descriptive adjectives/metaphors, etc. that might relate to what I was writing. Maybe even some suggested whole phrases’ (NS-06-N, woman, 48 years old).

*Overall evaluation*. Participants’ overall evaluation of the images was rather decisive: of the 19 participants who provided such an evaluation, 11 (57.89%) indicated their dislike and 9 (47.37%) indicated their neutrality (ex., ‘I neither liked nor disliked any of them’; NS-24-D, woman, 43 years old). While the participants took time to describe individual images that they liked more than others, only two participants mentioned that they liked (or, in one case, ‘didn’t mind’; NS-08-N, woman, 42 years old) the images as a whole. One of these individuals explained, ‘Although I liked the photos, the real photos were going on inside my head’ (NS-17-N, woman, 63 years old), indicating that the images were, to some extent, superficial. Responses to the application were more positive: of the 47 individuals who provided such an evaluation, 17 (36.17%) found the application to be sufficient (nothing more to add) or promising, while 11 (23.40%) indicated that they liked the application or its layout, with five (10.64%) indicating that they would use it again. Respondents particularly prized the motivating and streamlining aspects of the tool, which helped them to focus their thoughts on specific details in the story. Fifteen of the 47 individuals (31.92%) regarded the app as insufficient for story writing, indicating that they would not use the tool again (at least barring substantial changes), though only three (6.38%) explicitly stated their dislike for the application. Thirteen participants (27.66%) did not see much difference between the application and other writing tools, while 11 (23.40%) did not feel any particular way about the application or their experience using it. Some users envisioned interesting use cases for the application; one in particular stated that the app could help individuals with aphantasia—an inability to form mental images—to make their creative processes more visual. Although many participants commented on the distracting quality of the application or its features, and a few reported actual or anticipated therapeutic benefits, none envisioned a use case for the application that involved emotion regulation.

## 6. Discussion and conclusion

In this paper, we reported the development and user evaluation of StoryWriter, an AI-enhanced writing application that uses Dynamic Memory-Generative Adversarial Networks to generate real-time images from users’ written stories. These images are intended to serve as positive distractions that mitigate the impact of negative emotions in online writing activities. Based on our experimental study (Study 1) with 388 users and two control conditions (neutral emotion versus negative emotion and no image generation application versus StoryWriter application), we found that:

(i) users who were assigned to StoryWriter and/or induced with a negative emotion exhibited lower post-study anger and sadness than those in the control conditions, but not lower anxiety or stress,
(ii) users were somewhat successful in downregulating negative emotions through the application, but not in upregulating positive emotions (indicating certain boundary effects),
(iii) users induced with a negative emotion wrote significantly more characters and spent more time writing than those in the control condition.

While these results appear to support the usage of GAN-based image generation as a tool for emotion regulation, Study 2—our qualitative study carried out with 54 users—yielded somewhat different insights:

(1) Though users with pre-existing negative emotions did seem to be at least somewhat distracted from these emotions when using the application, the distraction was not consistently positive, and there was not much of an effect for induced escalated emotions.
(2) Though users in Study 1 did not exhibit substantial positive emotion upregulation, some users in Study 2 did exhibit some improvements in certain positive emotions—namely, those with a bias towards action.
(3) Furthermore, increased usage of the application did not necessarily mean that users were positively engaged with all (or even target) features of the StoryWriter application. Indeed, users appeared to attribute emotion regulatory benefits to the writing task, but not to the image generation feature.
(4) Independent of other safety indicators, many participants found the images to be grotesque and unsettling, even to the extent that some participants avoided looking at them.

Taking these findings together, and coupled with the lack of positive appraisals of the application’s images, we find that even if StoryWriter was successful in distracting participants (an outcome that applied to only about half of the participants in Study 2), the emotion regulatory mechanisms it facilitates appear unstable and driven by the fictional writing (therapy) rather than the concurrent image generation. Furthermore, the negative appraisals of the images indicate the model’s failure to safely strengthen emotional outcomes. Yet it also demonstrates the potential of the technology, in that the generated images leave a strong impression on the writer. Instead of deploying StoryWriter as a writing therapy tool, future studies may consider how such real-time visual feedback can be used by writers—especially those who may have difficulty with imagination—to help visualize their scenes and scenarios in real time.

Though our initial goal was to develop an application that generates images from users’ text input in order to facilitate emotion regulation, we find that StoryWriter has only a limited effect in alleviating negative emotions during expressive writing exercises, though it could enhance engagement in ways that support therapeutic outcomes. Our study also demonstrates how quantitative and qualitative approaches can be combined for a thorough analysis of user evaluations and feedback to new technologies.

Given our theoretical approach, does the limited effect of StoryWriter imply that positive distraction was ineffective in facilitating emotion regulation? Qualitative evidence from Study 2 suggests that for a majority of users who experienced an upregulation in their affective state, these were attributed to the writing process, instead of the generated images. One possibility could be that, for some individuals, the writing task (imaginative fictional writing) could be sufficiently distracting from their existing negative emotional state, thus facilitating positive emotional change. This would be a departure from standard autobiographical writing therapies (see Background), in that fictional narrative writing could provide a relatively safer environment for online writing therapies. Accordingly, our image generation should facilitate, rather than hinder or draw attention away from, the writing process, which we discuss in more detail in the following section.

### 6.1. Limitations

There were several limitations present in our studies which should be noted. Firstly, StoryWriter was designed for English-speaking users, relying on a GAN image generator trained on English language annotations. Our study also recruited (predominantly) American users with mostly native English language proficiency. As such, we have yet to evaluate the application in cultures and languages outside of the English-speaking US. Because StoryWriter’s textual input is fictional and anonymous, not demanding the disclosure of personal, traumatic incidents (as in conventional expressive-writing-based therapies), we expect that the tool will be particularly accessible to individuals from East-Asian countries, who are often uncomfortable with self-disclosure [52,65]. More research is recommended to appraise the tool’s efficacy for emotion regulation outside of Anglo-American contexts.

One large limitation would be that StoryWriter currently uses the DM-GAN model trained on photographs. This resulted in the generated images giving (in some cases) a ‘*grotesque*’ form of reality, which negatively impacted some users and drew attention away from the writing task. Given that some users were also disgruntled by the images’ irrelevance for their story, one solution would be to redesign the user interface such that the generated images are given less prominence than the writing task. By placing less importance on the generated images, we hope that it could play a supporting role in guiding users to continue with the development of their story, instead of discouraging users from writing due to the incongruence of the images. Also, we propose that training an image generation model on a specified dataset of artwork or landscapes, using newer image generation technologies (such as VQ-GAN) with more advanced text-to-image systems, would reduce the abstractness of the generated images while producing more accurate representations of the text. Alternatively, this could also be achieved through image retrieval technologies, where images are retrieved from a database of pictures (rather than generated from scratch). This would allow for greater control over the type of images displayed, and image modification technologies (such as style transfer) could also be applied

Given prevailing circumstances and logistical constraints, we completed our qualitative evaluation remotely through a long-form, open-ended survey. However, in-person user tests may have yielded richer, more detailed information, while psychiatric supervision would have allowed the researchers to explore participants’ negative emotions in greater depth (in ways that cannot be safely replicated in an online, remote survey). Future investigations may also benefit from a longitudinal, user-diary-based approach, allowing participants to use the application over a number of weeks and to record their impressions in a free-form way, alongside brief, periodic interviews.

## Data Availability

Anonymized participant data are available online at our Open Science Framework (OSF) repository (https://osf.io/6cpjv/).

## Footnotes

1
https://vuejs.org/

2
https://flask.palletsprojects.com/en/2.0.x/

3
https://prolific.co
